# Cyclitols and Aminocyclitols as Signals: Their Roles in Supporting Ecosystems and Promoting Human Health

**DOI:** 10.1021/acs.jnatprod.5c01437

**Published:** 2026-02-04

**Authors:** Leigh Skala, Patrick K. Murdach, Taylor Hennelly, Taifo Mahmud

**Affiliations:** Department of Pharmaceutical Sciences, 2694 Oregon State University, Corvallis, Oregon 97331-3507, United States

## Abstract

Secondary metabolites (a.k.a. natural products) are believed to play beneficial roles for the producing organisms, such as coping with environmental stress or outcompeting other organisms. Some of them may function as signals. One such class of natural products is the cyclitol/aminocyclitol family of compounds, which includes *myo*-inositol and its derivatives, the aminoglycoside antibiotics, and the C_7_-cyclitols and C_7_N-aminocyclitols. While the function of inositol and its derivatives as intracellular signaling molecules has been well established, more recent studies have also demonstrated their important ecological roles. Studies have also shown that aminoglycoside antibiotics may function as signals that regulate biofilm formation in bacteria and secondary metabolite production in fungi. Several C_7_-cyclitols and C_7_N-aminocyclitols, *e.g*., acarbose, validamycin, and kirkamide, have been associated with gut microbiota modulation, gene regulation in fungi and insects, and/or plant–bacteria endosymbiosis. While mycosporine-like amino acids (MAAs) are well known for their UV-protective activities, studies with human cells and animals have shown intriguing biological activities involving gene regulation and activation of signaling pathways. This article reviews the roles of this family of natural products as signaling molecules involved in various biological events, including bacterial colonization and gene regulation, with physiological and ecological implications that may affect human health and other organisms.

## Graphical abstract

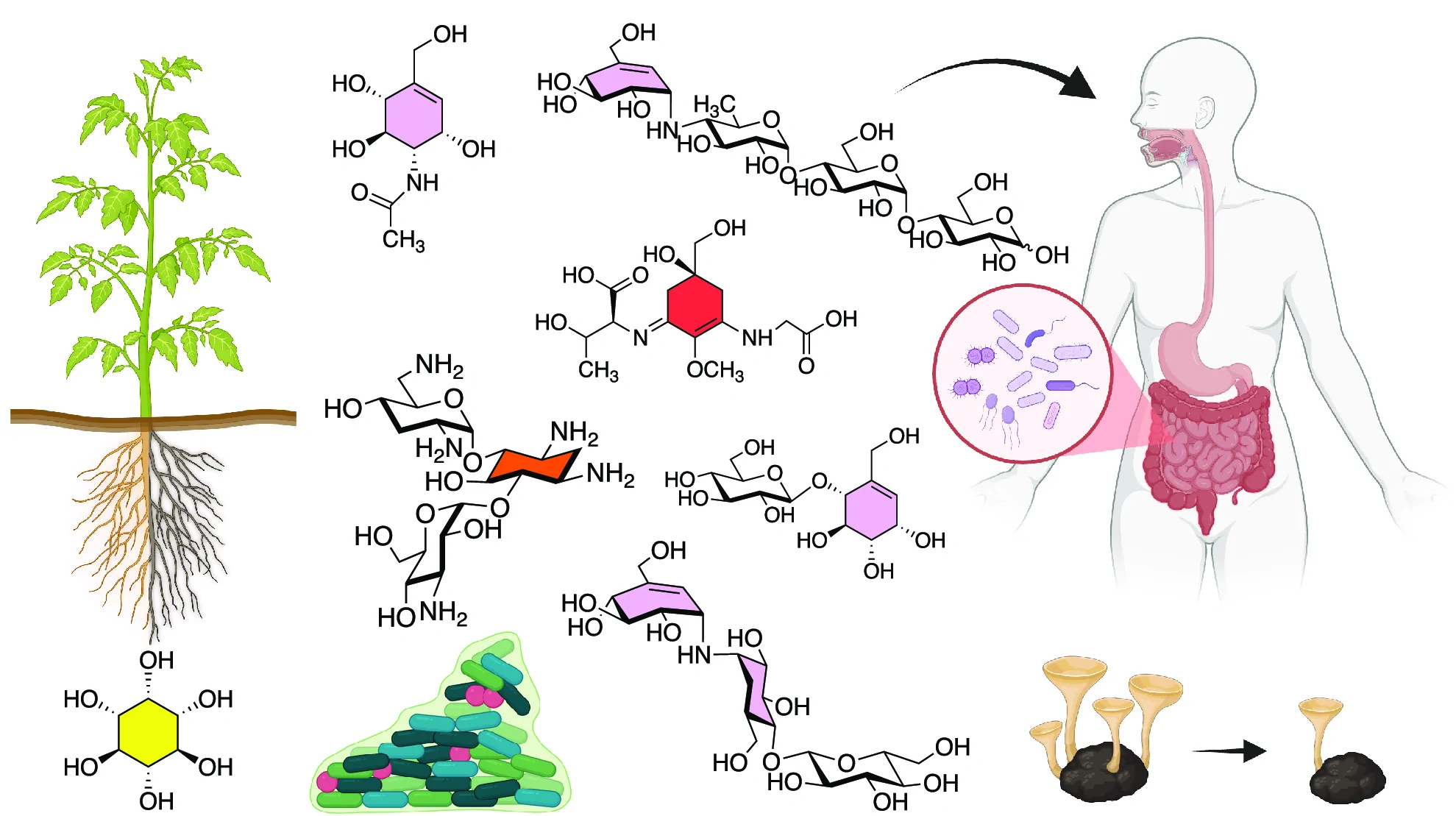

## Introduction

Organisms are known to produce a myriad of compounds that are not directly involved in their growth and reproduction but are sometimes essential for their survival in nature. These so-called secondary metabolites or natural products often have a beneficial role for the producer to help them cope with environmental stress or outcompete other organisms.^1^ Some of them may function as signaling molecules that are involved in quorum sensing or play a role in gene regulation. One such class of compounds is the cyclitol–aminocyclitol family of natural products, which are characterized by their cyclic alcohol structures (Figure 1).^2,3^ These include *myo*-inositol (**1**) and its derivatives, the aminoglycoside antibiotics [*e.g*., streptomycin (**2**) and tobramycin (**3**)], and the C_7_-cyclitols and C_7_N-aminocyclitols [*e.g*., acarbose (**4**), validamycin A (**5**), kirkamide (**6**), gadusol (**7**), and porphyra-334 (**7**)].

**Figure 1:**
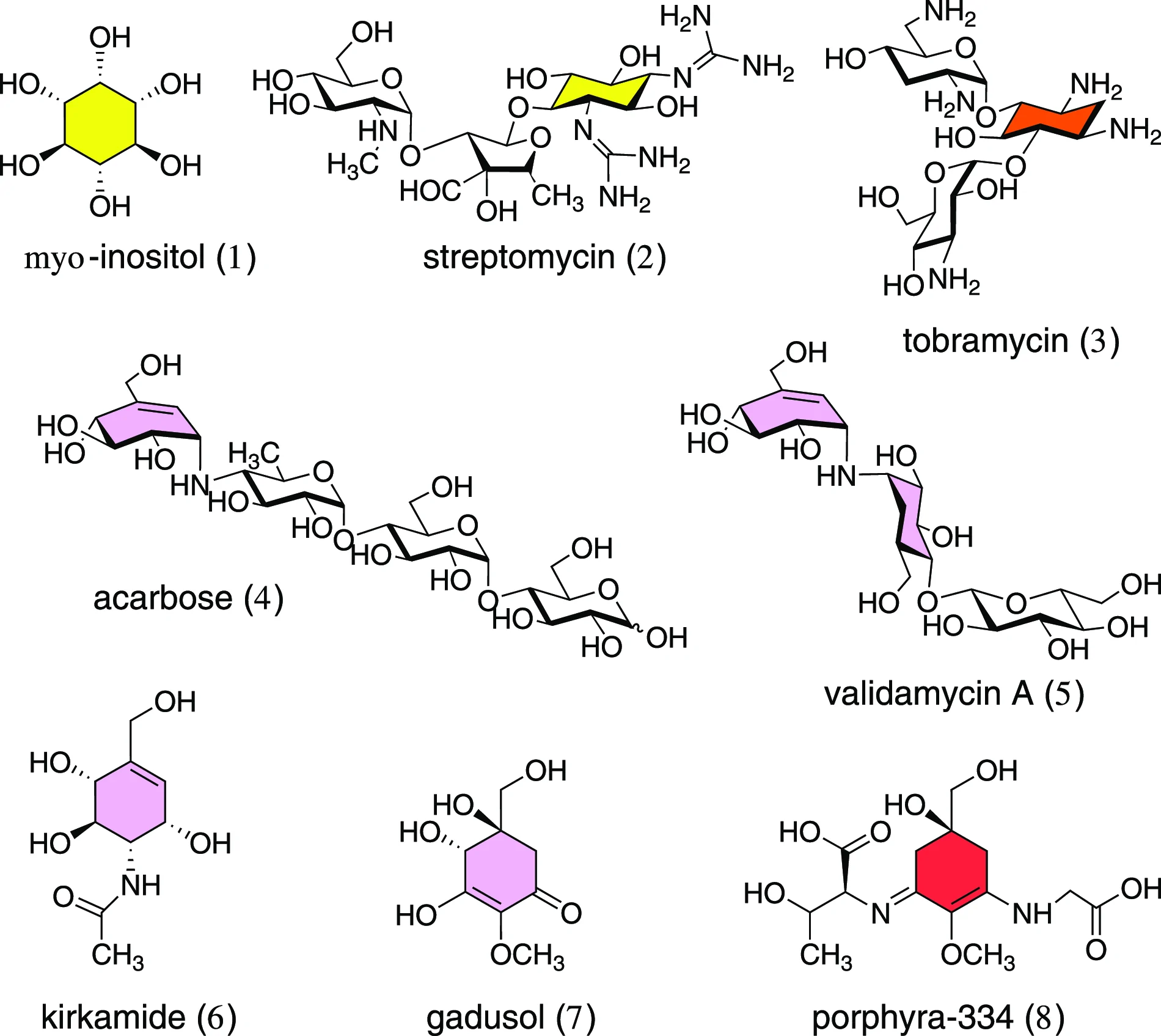
Chemical structures of several cyclitols–aminocyclitols. Different ring colors represent their distinct biosynthetic origins or pathways as follows: yellow = *myo*-inositol; orange = 2-deoxy-*scyllo*-inosose; pink = 2-*epi*-5-*epi*-valiolone; and red = desmethyl-4-deoxygadusol.

Biosynthetically, the above-mentioned cyclitols/aminocyclitols are derived from sugar phosphates (*e.g*., glucose 6-phosphate or sedoheptulose 7-phosphate) whose cyclization is catalyzed by sugar phosphate cyclase enzymes (SPCs) (Figure 2).^4,5^ *Myo*-inositol (**1**) is derived from glucose 6-phosphate through the catalysis of two enzymes, *myo*-inositol 1-phosphate synthase and *myo*-inositol 1-phosphatase.^5–7^ While some aminoglycoside antibiotics (*e.g*., streptomycin (**2**)) are derived from *myo*-inositol,^8,9^ the majority of aminoglycoside antibiotics are derived from 2-deoxy-*scyllo*-inosose (DOI), which is also a cyclic product of glucose 6-phosphate but catalyzed by a different enzyme, 2-deoxy-*scyllo*-inosose synthase (DOIS).^10^ The product (DOI) is then process through multiple step reactions to produce various aminoglycoside antibiotics.^3^ The precursors of the C_7_-cyclitols/C_7_N-aminocyclitols, 2-*epi*-5-*epi*-valiolone (EEV) and 2-*epi*-valiolone (EV), are both derived from sedoheptulose 7-phosphate, catalyzed by 2-*epi*-5-*epi*-valiolone synthase (EEVS) and 2-*epi*-valiolone synthase (EVS), respectively.^11–14^ These compounds are used to produce a wide variety of natural products, such as the antidiabetic drug acarbose (**4**), the antifungal agent validamycin A (**5**), the anti-insecticidal agent kirkamide (**6**), and the sunscreen compound gadusol (**7**).^15–17^ Sedoheptulose 7-phosphate is also a substrate of another SPC enzyme, known as desmethyl-4-deoxygadusol synthase (DDGS),^13,18^ to produce desmethyl-4-deoxygadusol (DDG). The latter is the precursor of the sunscreen compounds mycosporine-like amino acids (MAAs), *e.g*., porphyra-334 (**8**).^19,20^ The latest addition to the family of SPCs is 2-deoxy-4-*epi*-*scyllo*-inosose synthase (DEIS), which catalyzes the conversion of mannose 6-phosphate to 2-deoxy-4-*epi*-*scyllo*-inosose (DEI) (Figure 2).^21^ This enzyme is part of a cryptic biosynthetic pathway (the ebo pathway) found in bacteria and algae.^22–24^ While the final product of this cryptic pathway is currently unknown, the compound may function as a signaling or regulatory molecule in bacteria.^22,25,26^ Except for *myo*-inositol 1-phosphate synthase, the structures of the other SPCs described above are highly similar to that of 3-dehydroquinate synthase (DHQS) from the shikimate pathway.^19^

**Figure 2:**
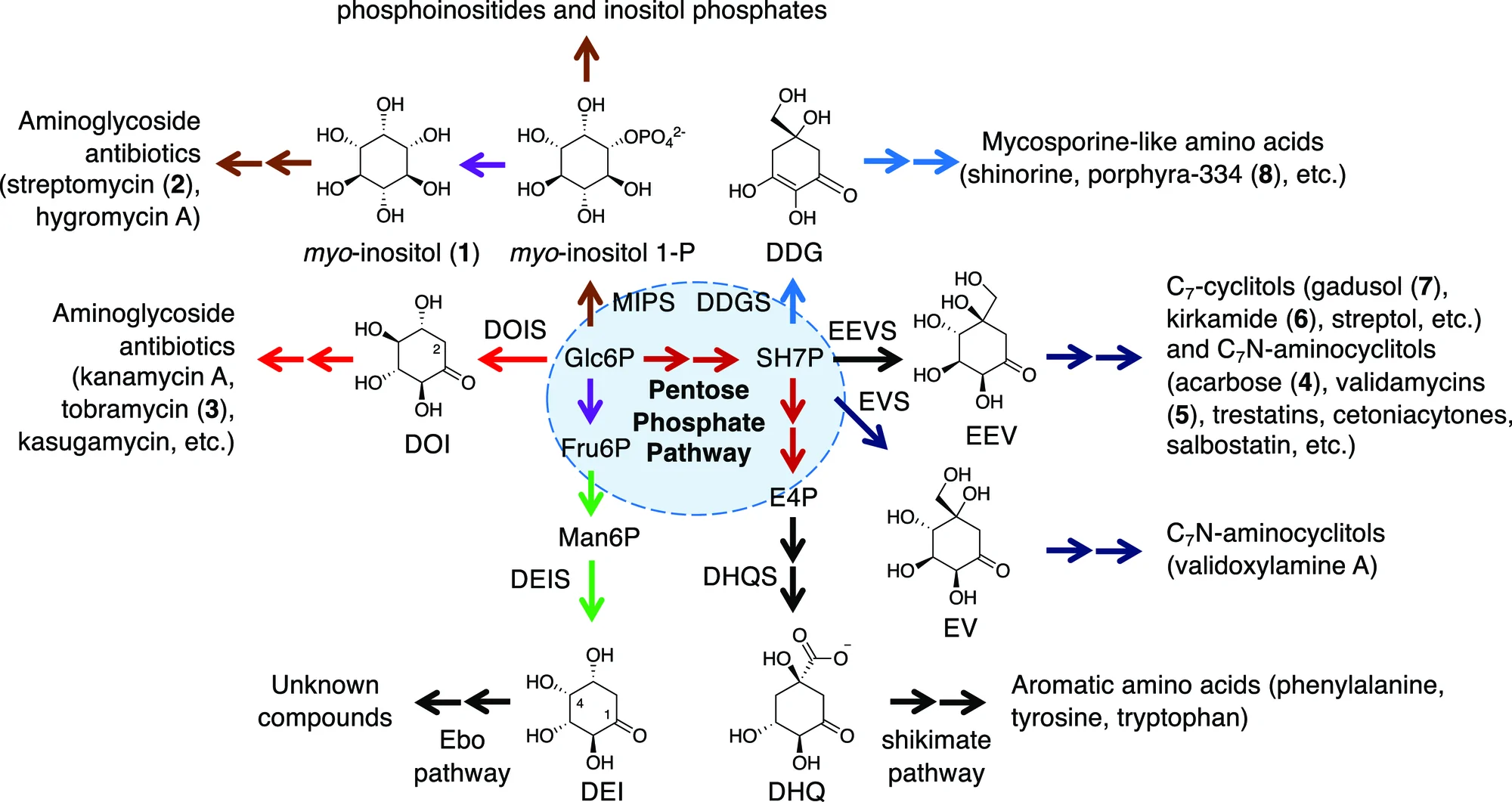
Formation of cyclitol- and aminocyclitol-derived natural products. MIPS, *myo*-inositol phosphate synthase; DOIS, 2-deoxy-*scyllo*-inosose synthase; DDGS, desmethyl-4-deoxygadusol synthase; EEVS, 2-*epi*-5-*epi*-valiolone synthase; EVS, 2-*epi*-valiolone synthase; DHQS, 3-dehydroquinate synthase; DEIS, 2-deoxy-4-*epi*-*scyllo*-inosose synthase.

The role of inositol and its derivatives (phosphoinositides and inositol phosphates) as intracellular signaling molecules has been well established in eukaryotic systems.^27,28^ In mammalian cells, they are involved in numerous cellular processes, *e.g*., membrane trafficking, signal transduction, and calcium dynamics.^28^ They orchestrate the spatial and temporal regulation of many signaling cascades, contributing to early life development as well as playing a role in metabolic homeostasis and the pathogenesis of age-related metabolic dysfunction and diseases.^27,28^ In plants, inositol derivatives also participate in signal transduction and are involved in calcium signaling, facilitate intercellular communication in stoma guard cells and roots, and contribute to the activation of stress-response genes.^29,30^ However, more recent reports also demonstrated important ecological roles of inositol in nutrient cycles as well as in plant’s host–endophyte interactions.^31,32^ Similarly, research data suggest potential ecological roles of the aminoglycoside antibiotics beyond their conventional niche protective function. Of particular interest is their function as signals that regulate biofilm formation in clinical and environmental bacteria.^33,34^ Some of them also regulate secondary metabolite production in fungi.^35,36^

Research evidence to support the roles of the C_7_-cyclitols or C_7_N-aminocyclitols in signaling and gene expression regulation have also begun to emerge. While the α-amylase inhibitor acarbose has been proposed to function as a competitive exclusion agent or a carbophore for the producing organisms,^37,38^ interesting studies related to the effects of this compound on gut microbiota and the downstream effects on human health have been reported more recently.^39–44^ Furthermore, reports on validamycin functions beyond its trehalase inhibitory activity, *e.g*., gene regulation in fungi and insects, are also emerging.^45–47^ Many other C_7_-cyclitols and C_7_N-aminocyclitols, *e.g*., kirkamide, streptol, and mycosporine-like amino acids, have been reported to play significant ecological roles, and some of which may function as signals.^48–53^ In this review, we explore the regulatory or signaling roles of the cyclitols and aminocyclitols in the environment and their implications for ecology and human health. As the field is still evolving and there are many gaps in knowledge related to this topic, we use “signal” as a loose term to include compounds that can affect gene expression and signal transduction, function as chemical cues, or are involved in symbiotic relationships. Consequently, some of the examples may not fit very well into the traditional definition of “signal” used in ecology or evolution. Nevertheless, we believe that some of the observed phenomena are intriguing and may serve as a useful starting point into studying cyclitols and their roles in supporting ecosystems and promoting human health.

## *Myo*-Inositol and Its Derivatives as Signaling Molecules

*Myo*-inositol (**1**) is a cyclic alcohol that is produced by various organisms, such as plants, animals, fungi, algae, and some bacteria.^54,55^ This compound was first isolated in 1850 from meat juice, and has been shown to have many biological functions.^7^ The role of *myo*-inositol (**1**) and its derivatives (*e.g*., phosphoinositides and inositol phosphates) as signaling molecules has been well established in eukaryotic systems.^27,28,55^ Many reviews on this topic are available in the literature;^27,28^ therefore, they will not be discussed in detail in this review article. However, *myo*-inositol may also play other roles in the environment, including acting as an osmotic regulator in aquatic organisms,^56,57^ enhancing drought tolerance in plants,^58,59^ and promoting plant-microbe symbiosis.^31,32,60,61^ While its property as an organic osmolyte and osmoprotectant has been well established, its role as a chemical cue in promoting plant-microbe symbiosis is fascinating and is elaborated in more detail below.

### *Myo*-Inositol in Plant-Microbe Symbiosis

Many plants establish symbiotic relationships with microorganisms to gain specific benefits, and this relationship can come in different forms. Plant endophytes (*e.g*., bacteria and fungi) provide many benefits to their host, such as promoting growth, tolerating stress, protecting the host from pathogens and pests, and/or helping the host to outcompete other plants.^62,63^ This is done by helping the host to acquire nutrients, modulate growth hormones, or fight off pathogens.^64^ For endophytes, motility and quorum sensing are major factors for successful colonization of a host.^64^ Interestingly, hosts have developed ways to help endophytes manage this feat using small molecule signals. Some plants release *myo*-inositol (**1**) through their roots to attract bacteria for colonization (Figure 3A).^32^ The *iol* gene cluster that is responsible for *myo*-inositol metabolism has been identified in 67% of *Pseudomonas* species which are known to be beneficial endophytes.^61,65^ Among them are *P. simiae* WCS417 and *P. protegens* CHA0, which displayed strong colonization of *Arabidopsis* roots grown in an axenic soil system.^61^ *Pseudomonas* isolates that lacked the complete *iol* gene cluster showed reduced colonization of the roots. Bacteria that harbor the *iol* gene cluster have the ability to metabolize *myo*-inositol and have better swimming motility than strains that do not have the cluster.^61^ In fact, only *P. protegens* CHA0 wild-type, not the Δ*iol* mutant, can grow in media with *myo*-inositol as the sole carbon source, albeit to only 50% the density reached with glucose. However, *myo*-inositol alone does not strongly enhance swimming motility. The activity was much more prominent when *myo*-inositol was added to a culture with glucose as the primary carbon source.^61^ Therefore, its activity to enhance bacteria motility and colonization seems to be much more than simply providing carbon to the bacteria.^61^ It may function as a signal for the plant bacterial colonization.

**Figure 3:**
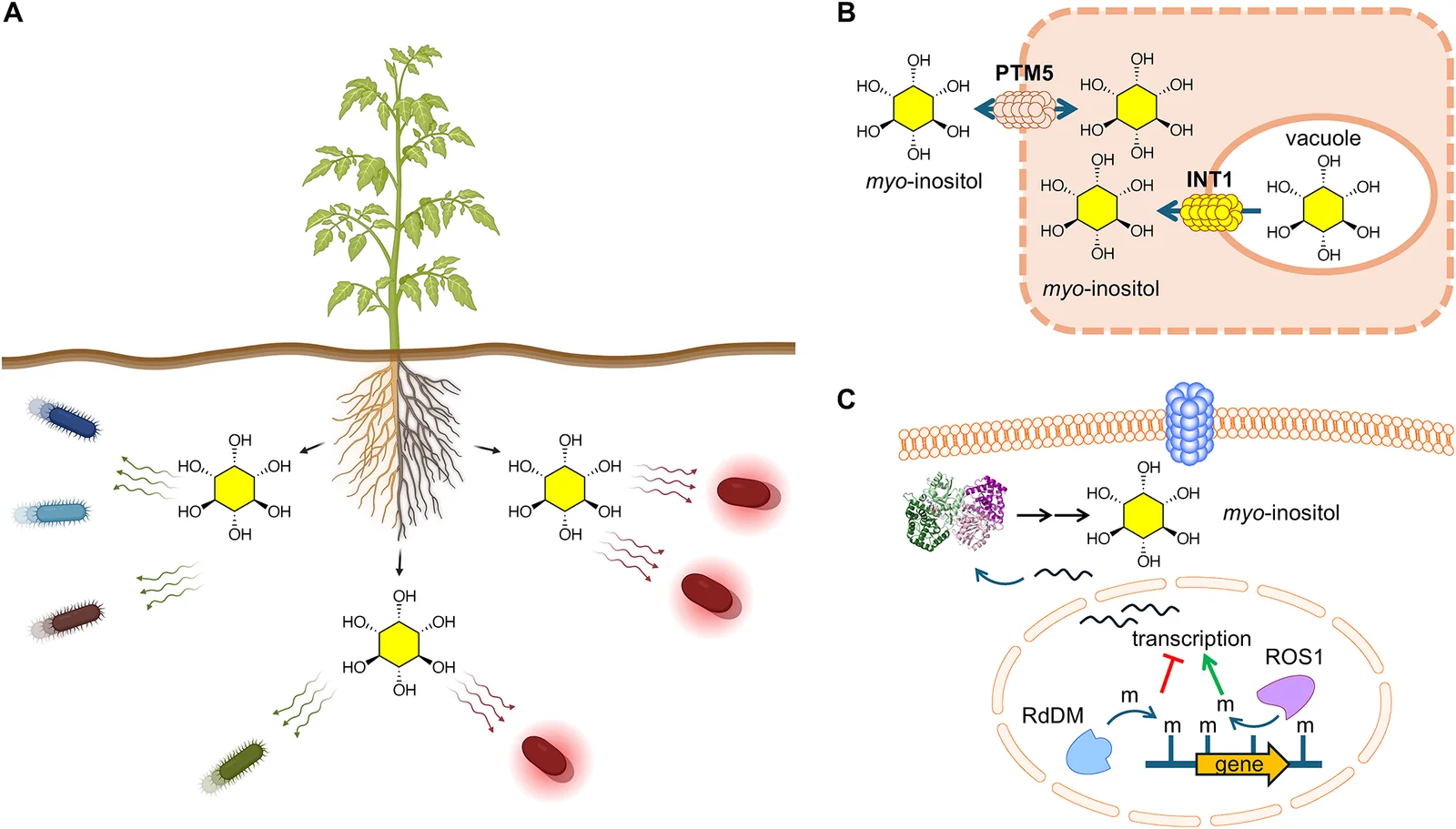
Plant *myo*-inositol promotes bacterial colonization. (A) Some plants release *myo*-inositol through their roots to attract bacteria for colonization (Created in BioRender. Hennelly, T. (2026) https://BioRender.com/5oidtjg); (B) a diagram showing the transport of *myo*-inositol across the tonoplast by INT1 and across the plasma membrane by PMT5; (C) secretion of *myo-*inositol controlled by synchronized DNA methylation/demethylation.

The importance of *myo*-inositol (**1**) as a signal or a chemical cue for bacterial colonization has also been demonstrated by altering the *myo*-inositol transporters in the host plant.^31^ Strains of *Pantoea* and *Streptomyces* were unable to effectively colonize the roots of *Arabidopsis* when the plant’s inositol transport has been disrupted.^31^ *Pantoea* has been associated with the *int1* gene, which encodes a tonoplast-localized transporter of *myo*-inositol (Figure 3B), as reduced root colonization was seen in *Arabidopsis* seedlings with *int1* disruption. On the other hand, *Streptomyces* was associated with the *pmt5* gene, which encodes a transporter of various polyols and monosaccharides (including *myo*-inositol) across the plasma membrane (Figure 3B). Disruption of this gene in *Arabidopsis* resulted in reduced colonization by *Streptomyces.* ^31^ This demonstrates the widespread utilization of *myo*-inositol as well as the importance of its transport by the plants.

Another study showed that *Arabidopsis* root secretion of *myo*-inositol (**1**) is controlled by active ROS1-dependent DNA demethylation, which, together with RNA-directed DNA methylation (RdDM), controls *myo*-inositol homeostasis (Figure 3C).^60^ This epigenetically controlled secretion of *myo*-inositol attracts *Bacillus megaterium* strain YCA and consequently promotes plant growth. The same DNA demethylation-dependent secretion mechanism of *myo*-inositol was also found to mediate mutualism between *B. megatarium* YC4 and tomato plants. In the presence of *myo*-inositol, *B. megatarium* expresses colonization-related genes at higher levels.^60^ Upon colonizing the roots, the bacterium promotes host growth through the production of siderophores and auxin.^61,66^ Siderophores produced by beneficial bacteria are known to supply iron to plants and help them grow.^67^ This type of plant-microbe symbiosis was also found to exist in other plants.^60^

However, the signaling molecule *myo*-inositol (**1**) does not always attract the intended crowd. *Ralstonia solanacearum*, a plant pathogen that causes lethal bacterial wilt disease of crops, is also attracted to *myo*-inositol (Figure 3A).^68^ Mutants of this pathogen lacking the ability to catabolize *myo*-inositol were unable to colonize plant roots.^68^ In low cell densities, this pathogen upregulates its *myo*-inositol catabolism pathways, and *myo*-inositol uptake seems to help better infection of roots at early stages.^68^ Consumption of other carbon sources is helpful for root infection only at higher cell densities or later stages of infection.^68^ Therefore, although *myo*-inositol may function as a chemical cue to attract bacteria toward plant roots, its catabolic intermediates (*e.g*., 2-keto-*myo*-inositol and 2,3-diketo-4-deoxy-*epi*-inositol) may be critical for the bacteria to infect and colonize the roots. Further studies in this area may provide better understanding of the role of *myo*-inositol in plant-microbe symbiosis.

## Aminoglycoside Antibiotics as Signaling Molecules

Aminoglycosides are a group of actinomycete-derived antibiotics characterized by the presence of an aminocyclitol core, mostly streptidine (**9**) or 2-deoxystreptamine (**10**), decorated with multiple sugar units (Figure 4).^69^ While streptidine (**9**) is derived from *myo*-inositol (**1**), 2-deoxystreptamine (**10**) is originated from 2-deoxy-*scyllo*-inosose (DOI, **11**).^3^ Aminoglycoside antibiotics bind to the bacterial 30S ribosomal subunit, specifically the 16S rRNA, and kill bacteria by interfering with their protein synthesis.^70^ Due to their strong antibacterial activity, the overarching ecological role of aminoglycosides has been to function as a weapon for its producer, to kill or outcompete other species of bacteria.^69,71^ However, studies have revealed that some aminoglycoside antibiotics are involved in regulating biofilm formation at subminimal inhibitory concentrations (SICs), as well as alter fugal secondary metabolite production.^33–36,72^

**Figure 4:**
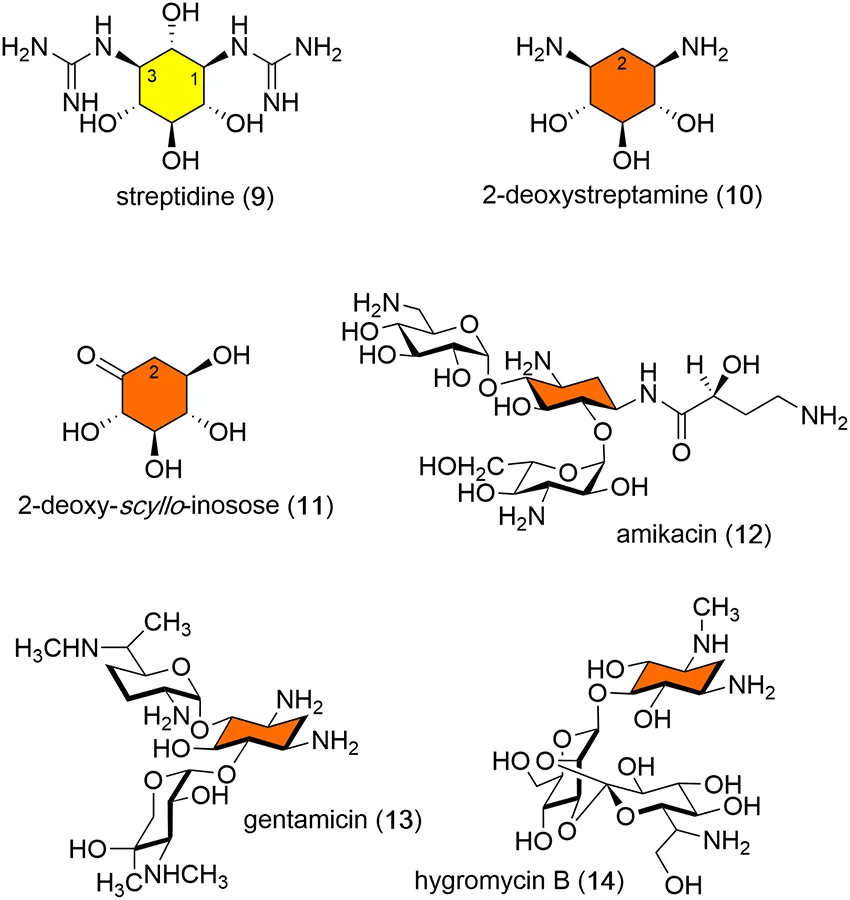
Chemical structures of several aminoglycoside antibiotics. The ring colored in yellow is derived from *myo*-inositol; the rings colored in orange are derived from 2-deoxy-*scyllo*-inosose.

### Tobramycin Induces Biofilm Formation

The aminoglycoside tobramycin (**3**), produced by *Streptomyces tenebrarius*, has been proposed to act as a signal in biofilm formation in *Pseudomonas aeruginosa*.^33,34^ Exposing the clinical isolate *P. aeruginosa* PAO1 to SICs of tobramycin (**3**) (less than a third of its MIC) increased its biofilm formation 3.4-fold than uninduced controls.^33^ Since *P. aeruginosa* and *S. tenebrarius* both dwell in soil, it has been proposed that *P. aeruginosa* naturally encounters tobramycin and consequently responds adaptively to aminoglycoside exposure by forming antibiotic-resistant biofilms.^33^ Other aminoglycosides such as amikacin (**12**), streptomycin (**2**), and gentamicin (**13**) (Figure 4) also showed the same effect on *P. aeruginosa* PAO1, but with less potency.^33^ The biofilm induction activity of tobramycin was also observed in *E. coli*, implicating a conserved signaling pathway in both organisms.^33^

Interestingly, opposite effects of tobramycin (**3**) was observed in the environmental isolate *P. aeruginosa* PUPa3 (Figure 5A).^72^ When the PUPa3 strain was exposed to tobramycin at SICs, its biofilm formation and swarming abilities were conversely reduced.^72^ In this strain, SICs of tobramycin affected two independent *N*-homoserine lactone quorum-sensing (AHL QS) systems, indicating that these systems may be directly involved in the reduction of biofilm formation.^72^ In contrast, low or no quorum sensing activity was observed in the PAO1 clinical strain when exposed to SICs of tobramycin.^73^ Instead, an aminoglycoside response regulator (*arr*) gene was found to be involved in modulating biofilm formation in PAO1 exposed to tobramycin (**3**).^33^ This gene is predicted to encode an inner-membrane phosphodiesterase that degrades cyclic diguanosine monophosphate (c-di-GMP) and controls its levels (Figure 5B).^33^ The latter compound is a bacterial secondary messenger that regulates cell surface adhesiveness, biofilm formation, motility, and virulence.^74^ Inactivation of the *arr* gene resulted in mutants with diminished c-di-GMP phosphodiesterase activity and defective biofilm response to tobramycin.^33^ Although one would expect that enhanced activity of Arr by tobramycin would reduce biofilm formation, in most *P. aeruginosa* strains, an opposite effect was observed. A reduced pool of c-di-GMP due to tobramycin-induced Arr activity seems to trigger an increase in biofilm formation through an unknown mechanism.

**Figure 5:**
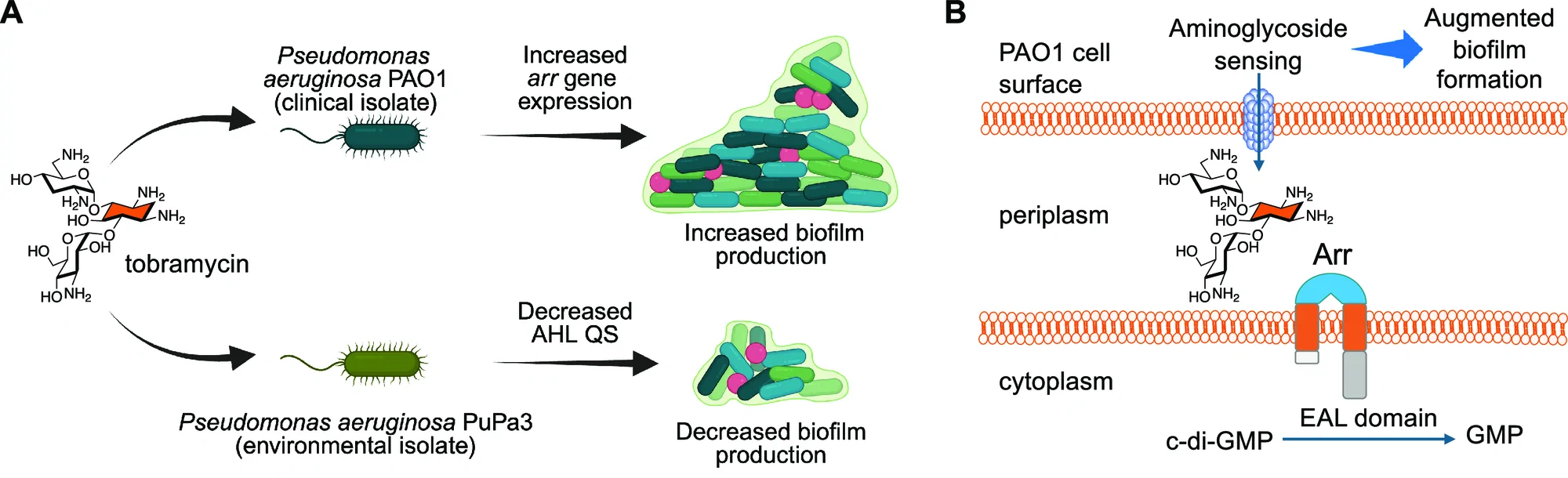
Tobramycin can act as a signal in biofilm formation in *Pseudomonas aeruginosa*. (**A**) Reported opposite effects of tobramycin in biofilm formation in different strains of *Pseudomonas aeruginosa*; (**B**) a model for tobramycin effects on *P. aeruginosa* PAO1’s biofilm formation involving the Arr protein (created in BioRender. Hennelly, T. (2026) https://BioRender.com/oftz5yw).

There were also conflicting reports on the mechanism underlying the increased formation of biofilm by *P. aeruginosa* PAO1 exposed to SICs of tobramycin. This increase was initially determined to be due to an increase in cell number, not in extracellular matrix.^33^ In addition, tobramycin was also found to inhibit flagellar motility and have no effect on type-IV pili-mediated twitching motility,^33^ leading to the notion that the induction of biofilm formation in PAO1 is not due to an increase in motility, but through a different mechanism involving *arr*. However, a subsequent study with the same strain showed that SICs of tobramycin did increase bacterial motility and biofilm formation,^34^ which is more consistent with the report that swarming and swimming are useful properties for aggregation and biofilm formation.^75^ Nevertheless, it has been suggested that tobramycin signaling can have different effects in different strains or when grown under different culture conditions.^72^

A more recent study using a combination of proteomics, transcriptomics, confocal laser scanning microscopy, and other phenotypic studies suggested a possible adaptive mechanism involving regulatory PrrF small noncoding RNAs (sRNAs), QS signaling, biofilm cell death, and extracellular DNA (eDNA) release in tobramycin-induced biofilm formation in *P. aeruginosa* H103.^76^ The release of eDNA, which is postulated to be modulated by the 4-hydroxy-2-alkylquinolines (HAQs) and regulatory PrrF sRNAs, is associated with the role of the biofilm matrix.^76^ Another study also reported that streptomycin increased biofilm formation by *Staphylococcus aureus* and *Pseudomonas aeruginosa* NRRL-B3509 by altering cell surface properties via upregulation of extracellular matrix-binding properties.^77^ However, numerous metabolic pathways appeared to be affected by tobramycin, suggesting a more complex mechanism is involved.^76^ While SICs of antibiotics in the environment have been proposed to function as signals, studies also showed that SICs of streptomycin are sufficient to inhibit competing susceptible *Streptomyces coelicolor* cells as well as to provide natural selection to enrich for resistance.^78^ Therefore, whether SICs of antibiotics in the environment are really being used as signals, weapons, or both remains a subject of debate.

### Hygromycin B Regulates Fungal Secondary Metabolite Production

In addition to inducing biofilm formation, some aminoglycoside antibiotics have been reported to function as inducers of secondary metabolite production in other organisms.^35,36^ The aminoglycoside hygromycin B (**14**) has been reported to induce secondary metabolite production in the filamentous fungus *Fusarium* sp. RK97-94.^36^ RNA-seq analysis of the fungus, cultured with and without hygromycin B (300 μg/mL), revealed the effects of hygromycin B at the transcriptional level. Seven out of the 42 predicted biosynthetic gene clusters within *Fusarium* sp. RK97-94 were found to be induced more than 2-fold by the antibiotic, including those of fusaribin (**15**), lucilactaene (**16**), and the polyketide 1233A (**17**) (Figure 6).^36^ The versatility and usefulness of this antibiotic as a broad-spectrum inducer of secondary metabolites were subsequently investigated using diverse fungal taxa (Eurotiomycetes, Dothideomycetes, Sordariomycetes), resulted in observed induction of secondary metabolite production in 20 out of 28 fungal strains tested (71%).^35^ The effect of hygromycin B (**14**) in secondary metabolite production in some of those strains was substantial, with the highest increase in production being 43-fold. The study also led to the identification of several novel natural products, hannocateol (**18**) and shirazines A and B (**19** and **20**) (Figure 6). The production of these compounds increased (up to >100-fold) with hygromycin B supplementation up to 100 μg/g (grown statically on water-sodden rice for 14 days). Interestingly, other eukaryotic protein synthesis inhibitors such as paromomycin (another aminoglycoside) and 5-fluorouracil also showed similar effects, albeit less consistently, suggesting that the underlying induction mechanism is associated with protein synthesis inhibition. Subsequent studies concluded that the induction of secondary metabolite production by hygromycin B (**14**) is not related to HDAC inhibition or due to drug-resistant mutations, which may lead to improved secondary metabolite production.^35^

**Figure 6:**
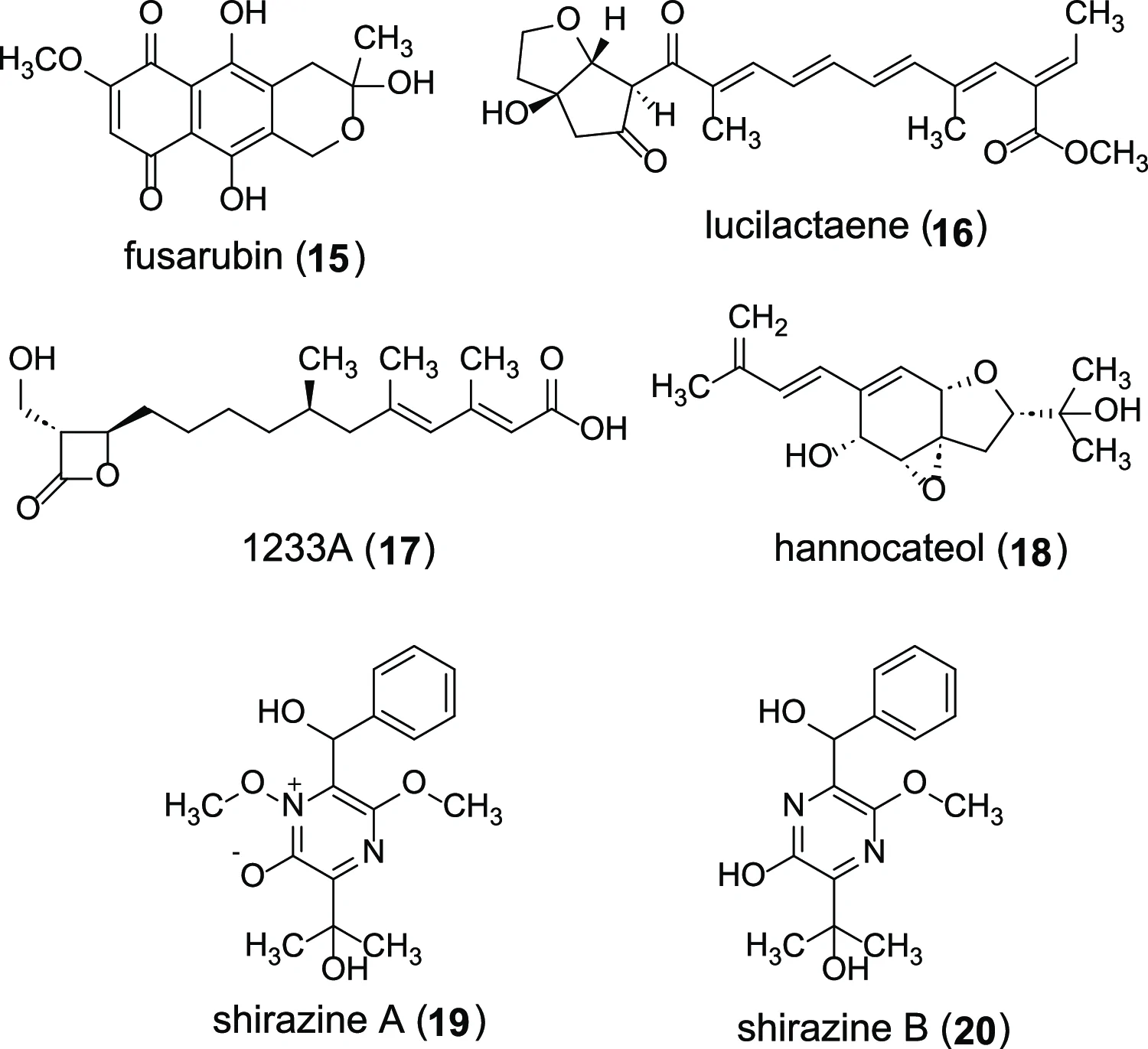
Chemical structures of new natural products whose production was upregulated by hygromycin B (**14**).

## C_7_-cyclitols and C_7_N-aminocyclitols as Signaling Molecules

In addition to the C_6_-cyclitol-containing compounds (*myo*-inositol and aminoglycoside antibiotics) described above, there are members of the cyclitol/aminocyclitol family of natural products that contain seven carbons in their core structures.^2^ These compounds are derived from a seven-carbon sugar phosphate, sedoheptulose 7-phosphate, an intermediate of the pentose phosphate pathway.^11,12,79^ This family of compounds is characterized by the presence of one or more C_7_-cyclitols or C_7_N-aminocyclitols in their structures, coming in many different configurations and shapes (Figure 7).^2^ The cyclitols can be part of oligosaccharides [dubbed as pseudo-oligosaccharides, *e.g*., acarbose (**4**), trestatins (**21**), validamycin A (**5**), validoxylamine A (**22**), and salbostatin (**23**)] (Figure 7). They can attach to different classes of natural products [*e.g*., pyralomicin (**24**), BE-40644 (**25**), porphyra-334 (**8**)]. The cyclitols can also form amides [*e.g*., cetoniacytones (**26**), epoxyquinomycin (**27**), kirkamide (**6**)] or are produced as simple monomers, such as gabosine A **(28**), gadusol (**7**), streptol (**29**) and its glycoside (**30**). This family of natural products is known to have various biological activities, ranging from α-glucosidase inhibition to antitumor to sunscreen.^80–82^ However, recent studies have also revealed their effects on microbiota or their functioning as signaling molecules that have broad implications in the environment and human health.^39–41,44,83^

**Figure 7:**
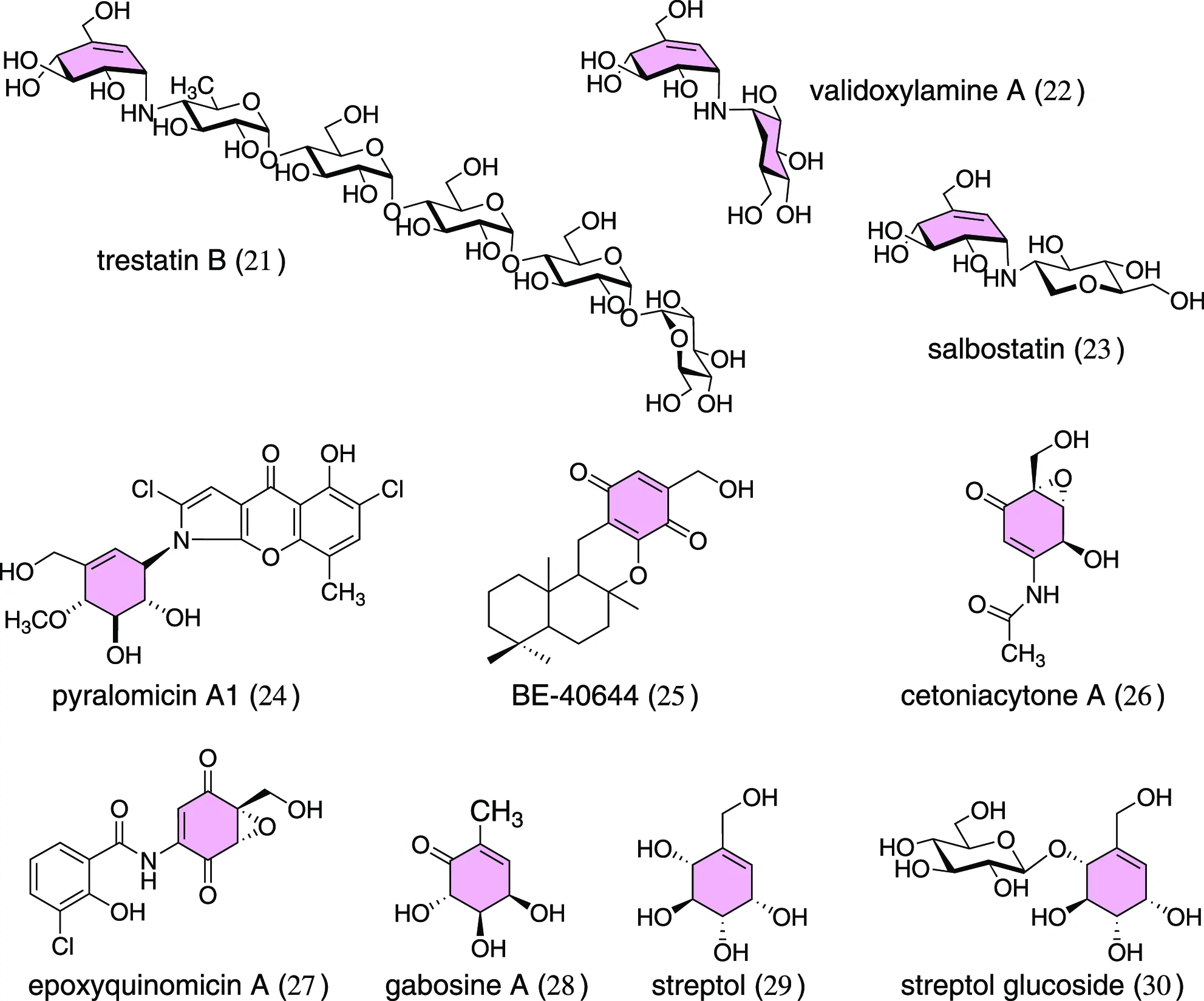
Chemical structures of C_7_-cyclitols and C_7_N-aminocyclitols. The rings colored in pink are derived from 2-*epi*-5-*epi*-valiolone.

### Effects of Acarbose on Gut Microbiota

Among members of the C_7_N-aminocyclitol family of natural products, acarbose (**4**) is arguably the most well studied compound. This natural α-glucosidase inhibitor was first isolated from *Actinoplanes* sp. SE50/110 and was developed to be a clinically used antidiabetic drug.^84,85^ Due to its strong α-amylase inhibitory activity, acarbose has been suggested to function as a competitive exclusion agent that is used by the producing organisms to compete with other bacteria for starches in their natural habitat (Figure 8A).^37^ Indeed, acarbose producing bacteria are equipped with acarbose-resistant α-glucosidases.^37^ In addition, numerous studies related to the effects of acarbose consumption on gut microbiota in both mouse models and humans have been reported (Figure 8B).^39–41^ Mice treated with acarbose had different microbial communities, increased concentrations of short-chain fatty acids (SCFAs) in the feces, and increased longevity, compared to the control.^41^ The increase in longevity has been correlated with the elevated production of SCFAs, which was strongly correlated with a bloom in members of the *Bacteroidales* family *Muribaculaceae*.^41^ By remodeling gut microbiota and altering the production of short-chain fatty acids, acarbose was also found to suppress symptoms of mitochondrial diseases in a mouse model.^44^ Interestingly, despite the positive correlation between acarbose and *Bacteroidales* population in general, acarbose impairs the growth of some species of gut *Bacteroides* but not the others by targeting their intracellular glucosidases.^42^ These differences appear to be due to the highly complex function and regulation of the starch utilization system in *Bacteroides,* including variation between species.^42^

**Figure 8:**
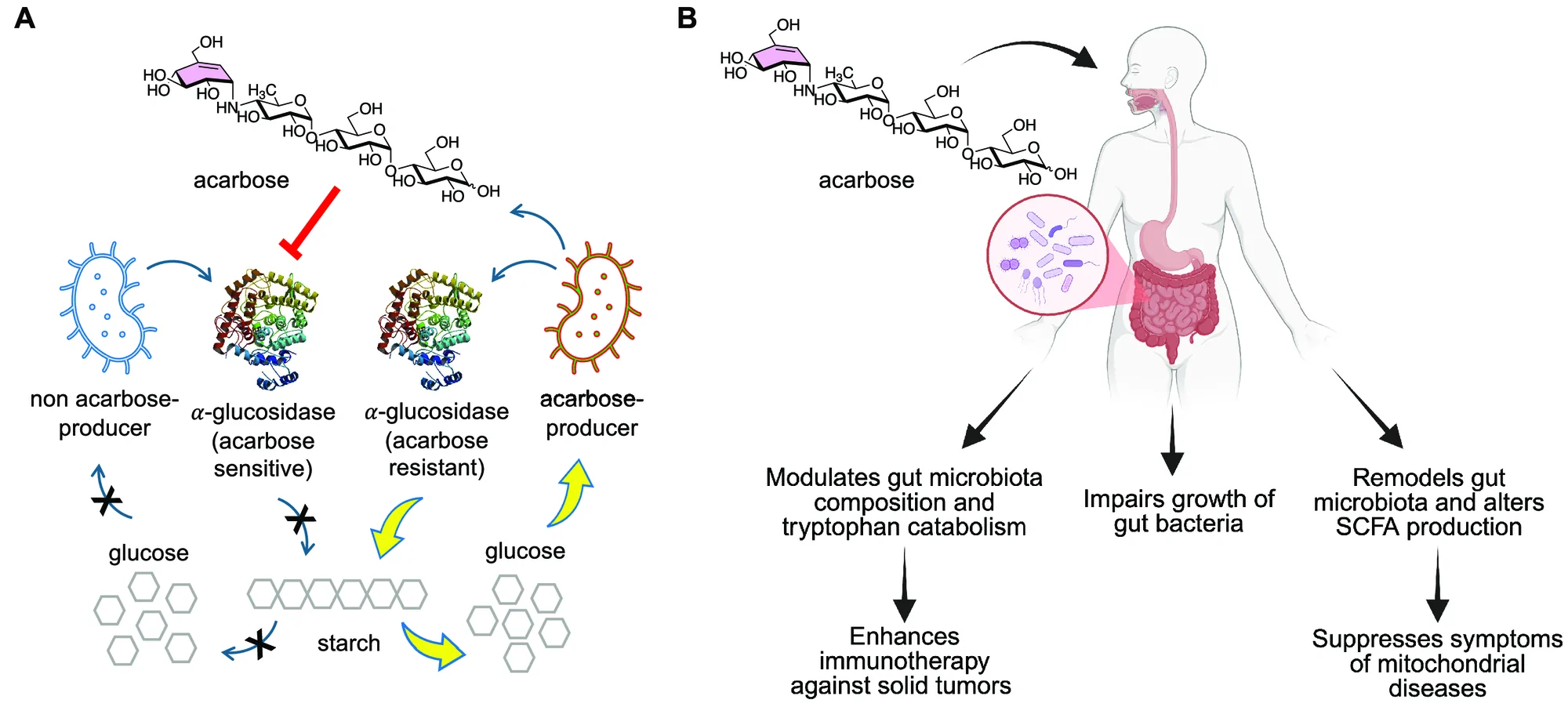
Effects of acarbose in microbiota. (A) Acarbose may function as a competitive exclusion agent that inhibits α-glucosidases from acarbose-nonproducing bacteria. α-Glucosidases from acarbose-producing bacteria are resistant to acarbose inhibition; (B) acarbose can modulate gut microbiota and impair the growth of gut *bacteroides*, resulting in various biological outcomes in humans and/or test animals (created in BioRender. Hennelly, T. (2026) https://BioRender.com/x6sfqqx).

By modulating the gut microbiota composition, acarbose also modulates tryptophan catabolism, resulting in enhanced efficacy of immunotherapy against solid tumors.^43^ Mouse gut microbiota treated with anti-PD-1 therapy (an immune-checkpoint inhibitor) were dominated by Firmicutes, whereas those treated with anti-PD-1+acarbose were dominated by Bacteroidetes. However, species from other phyla, such as Tenericutes, Actinobacteria, Proteobacteria and Verrucomicrobia, were also enriched in the acarbose-treated group. A combination of untargeted and targeted metabolomics and gene coexpression network analysis reveals the association of acarbose treatment with elevated activity of biochemical pathways related to tryptophan and several other amino acid metabolisms.^43^ Most significantly was the enrichment of indoleacetate (a tryptophan catabolite), which was found to have positive correlations with CD8^+^ T cell-associated chemokines, such as CXCL9 and CXCL10.^43^ In fact, the use of external indoleacetate did significantly enhanced the effect of anti-PD-1 therapy in inhibiting tumor growth in mice.^43^

A broad population-based, matched case-control study involving 7,230 diabetes mellitus (DM) patients found that regular use of acarbose (>16,950 mg per year) lowered the rheumatoid arthritis (RA) risk of diabetic patients.^86^ Prophylactic treatment of acarbose in arthritis-induced mice also showed a significant decrease in the production of antichicken CII antibodies and certain cytokines (*e.g*., IL-2, IL-6, IL-9, IL-17), as well as the histopathological severity of arthritis compared to control mice.^40,86^ In addition, acarbose reduced the Th17 cell frequency in the mice intestinal lamina propria and increased CD4^+^CD25^+^Foxp3^+^ T-regulatory (Treg) cells with elevation of Helios and CCR6. Interestingly, most of these immunomodulatory effects were not observed when acarbose was given at the time of arthritis induction.^40^ As in other studies, these changes have also been linked to the alteration in microbial community due to the α-glucosidase inhibitory activity of acarbose.^40^ Interestingly, miglitol, a related α-glucosidase inhibitor, showed similar but less consistent results. It also showed much less potent antiarthritic effect than acarbose.^40^

Changes in the composition of gut microbiomes due to acarbose intervention may also be linked to the habitual dietary intake or the intake of different staple foods.^83^ For example, the amount of change (delta) in the genus *Faecalibacterium* was positively correlated with the intake of rice normally found in Asian diet, but negatively correlated with the intake of bread. The intake of potato was positively correlated to the relative abundance of *Bacteroides* delta but negatively correlated with the variation of *Akkermansia* and *Subdoligranulum* genera.^83^

While most of the studies to date have associated the various benefits of acarbose to the alteration of gut microbiota through its α-glucosidase inhibition, there is no direct evidence to suggest that the observed physiological outcomes or the remodeling of gut microbiota was solely due to its α-glucosidase inhibitory activity. Moreover, some gut bacteria can degrade or modify acarbose to its phosphorylated products.^87,88^ A gut acarbose-degrading bacterium, *Klebsiella grimontii* TD1, which is widely distributed in human intestinal microorganisms, has been found to degrade acarbose into small molecules.^87^ Although chemical modifications may render it inactive as an α-glucosidase inhibitor leading to acarbose resistance in patients,^87,88^ the degradation products, *e.g*., smaller aminocyclitols, may possess other functions.

### The Role of Validamycin in Trehalase Inhibition and Beyond

Another well-studied member of the C_7_N-aminocyclitol natural products is validamycin A (a.k.a. jinggangmycin) (**5**), an antifungal agent originally isolated from *Streptomyces hygroscopicus* var. *limoneus* and later from the subspecies *jinganggensis* 5008.^89^ Structurally, validamycin A contains validoxylamine A (**22**) (which consists of two C_7_-cyclitol units connected by a nitrogen bridge) and glucose (Figure 7). While **22** is the active aminocyclitol pharmacophore, the attachment of glucose (the complete form) is necessary for the compound to enter fungal mycelia.^90^ Validoxylamine A (**22**) is also produced by *Actinosynnema mirum* DSM 43827, but through a slightly different biosynthetic pathway.^14^

Commercially, validamycin A (**5**) is used as a crop protectant against *Rhizoctonia solani* (syn. *Thanetophorus cucumeris*, *Pellicularia sasakii*), a sheath blight-causing fungus that infects rice plants. This natural product acts as a trehalase inhibitor, inhibiting the activity of the enzyme responsible for trehalose degradation.^90,91^ This in turn inhibits hyphal extension in *R. solani*.^92^ However, the trehalase inhibitory activity appears to have many environmental implications as well, ranging from improving plant responses to salt stress to delaying the development and preventing flight in insects.^93,94^ By inhibiting trehalase, validamycin A increases the concentration of trehalose (an osmoprotectant against salt stress conditions) in root nodules of *Medicago truncatula*.^93^ Since trehalase is required for the regulation of metabolism and glucose generation in insects, inhibition of trehalase by validamycin A also profoundly affects their physiology and morphology. For example, exposing *Aedes aegypti* mosquito with validamycin A delays its larval and pupal development and prevents flight of adult mosquitoes.^94^ The larvae were hypoglycemic, and the pupae had increased levels of trehalose, consistent with validamycin A’s activity as a potent trehalase inhibitor.

However, reports on validamycin functions beyond its trehalase inhibitory activity have started to emerge in recent years. Transcriptome analysis in *Rhizoctonia cerealis* treated with validamycin revealed downregulation of genes that are involved in metabolic processes, ribosome biogenesis, and pathogenicity.^45^ The data also showed that validamycin affected the expression of genes related to the MAPK signaling pathway, leading to decreased ribosome synthesis and assembly, and the suppression of the fungal growth (Figure 9). Effects of validamycin on gene regulation was also associated with validamycin suppression of reproduction in the small brown planthopper, *Laodelphax striatellus* (Fallen).^46^ Exposure to validamycin A led to reduced egg-laying by 47%.^47^ It was proposed that validamycin mediates downregulation of glucose dehydrogenase and l-3-hydroxyacyl-coenzyme A dehydrogenase (LCHAD) leading to the suppression of reproduction in small brown planthopper (Figure 9).^46,47^

**Figure 9:**
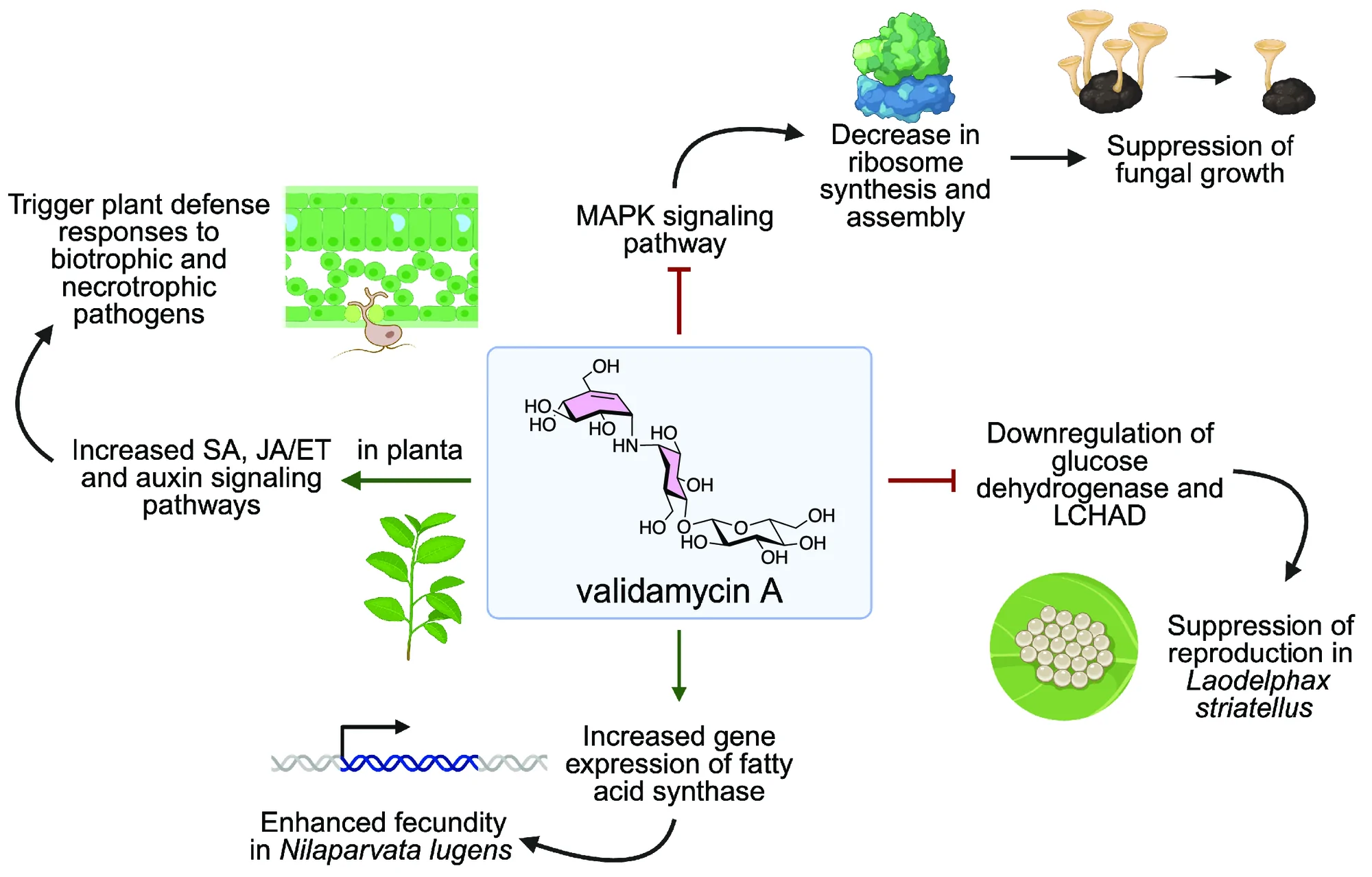
Regulatory effects of validamycin A (Created in BioRender. Hennelly, T. (2026) https://BioRender.com/p8be0ie).

Interestingly, while validamycin A suppressed the reproduction of small brown planthopper, it enhanced the reproduction of the brown planthopper *Nilaparvata lugens*.^95^ Quantitative analysis using iTRAQ protocols of ovarian proteins of brown planthopper females following validamycin foliar spray or topical application revealed changes in the expression of 250–300 proteins in validamycin-exposed groups compared to the controls. Specifically, increased fatty acid synthase gene expression in the treated groups has been correlated with the validamycin-enhanced fecundity in brown planthoppers, with increased gene expression potentially providing needed fatty acids for reproduction.^95–97^

Validamycin A has also been reported to trigger plant defense responses to both biotrophic and necrotrophic pathogens via salicylic acid (SA) and jasmonic acid/ethylene (JA/ET) signaling pathways.^98^ Transcriptome analysis revealed its effects on the expression of genes related to SA, JA/ET, abscisic acid (ABA), and auxin signaling pathways (Figure 9).

### The Roles of Kirkamide and Streptol in Symbiosis

Another C_7_N-aminocyclitol important for plant-bacterium symbiosis is kirkamide (**6**), which is produced by the obligate symbiont “*Candidatus* Burkholderia kirkii” (“*Ca*. B. kirkii”).^48^ The symbiosis between “*Ca*. B. kirkii” and its host plant, *Psychotria kirkii*, was found to be vertically transmitted, suggesting an extreme reliance between the host and the symbiont.^99–101^ In fact, kirkamide (**6**) has potent insecticidal activity, with a 90% mortality rate among pollen beetles exposed to the compound (Figure 10). Consequently, it has been proposed to have a protective role in the *Psychotria*/*Burkholderia* leaf nodule symbiosis.^48^ The relationship between the aminocyclitol producing biosynthetic gene cluster (BGC) in “*Ca*. B. kirkii” and *P. kirkii* has been extensively studied through genomic sequencing approaches. Genotyping of “*Ca*. B. kirkii” revealed that the EEVS gene involved in kirkamide biosynthesis is present in a plasmid, pKIR01.^102^ The kirkamide BGC (located on the plasmid) and the natural product, are present in a variety of other plant symbiotic *Burkholderia* species,^100^ highlighting the benefit of this compound to the organisms. This conservation of the kirkamide BGC across several *Burkholderia* species was suggested to be due to horizontal gene transfer,^100^ and the fact that it has withstood genome erosion may be due to the beneficial role of kirkamide (**6**) to the plant.^103^

**Figure 10:**
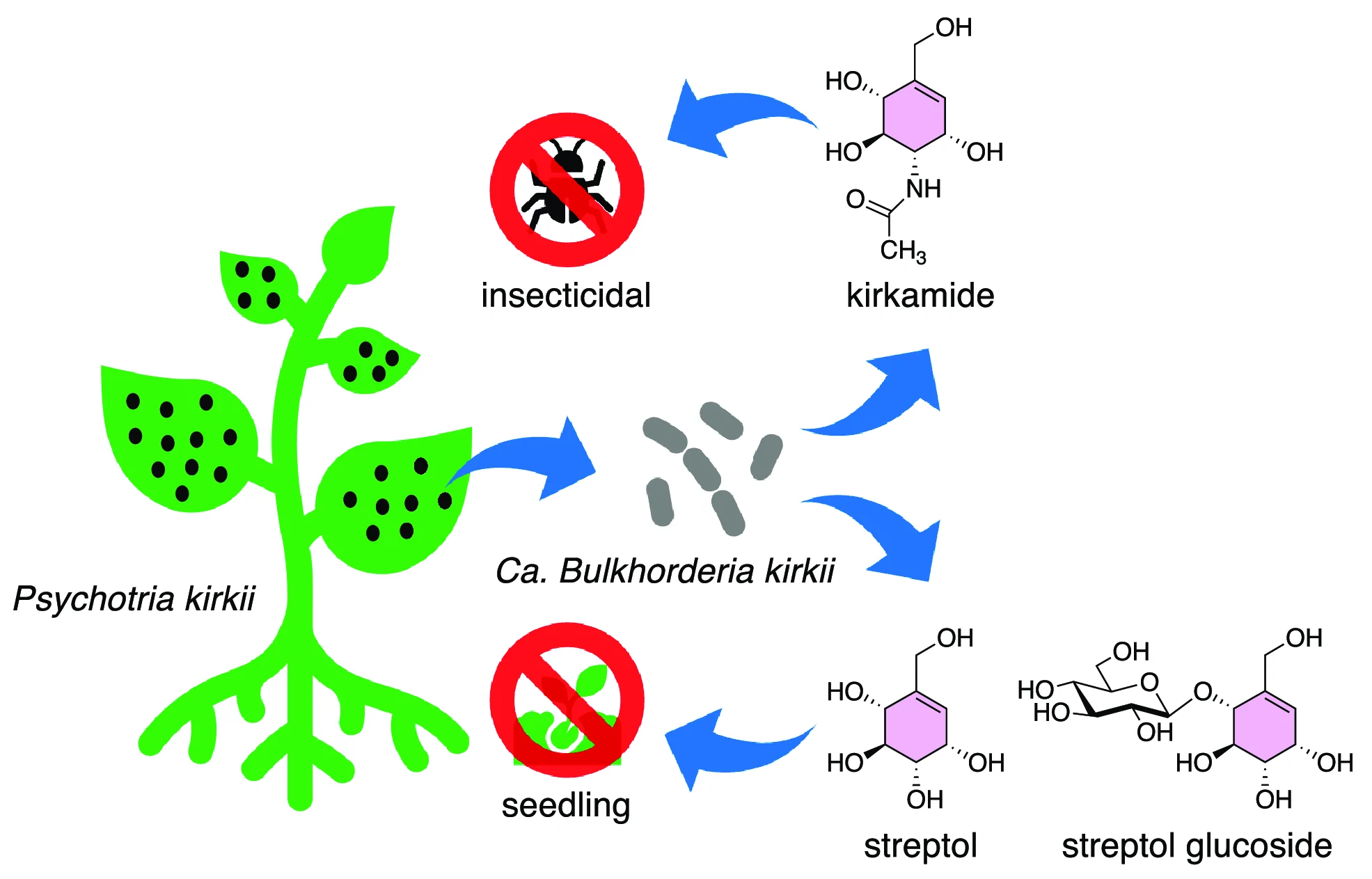
Roles of kirkamide and streptol in plant-bacterium symbiosis.

In addition to kirkamide (**6**), related C_7_-cyclitols [streptol (**29**) and streptol β-glucoside (**30**)] were also identified in “*Ca*. B. kirkii” (Figure 10). (+)-Streptol (**29**) was first isolated from an unidentified strain of *Streptomyces* and identified as a plant growth inhibitor (>13 ppm) and was lethal at a concentration above 50 ppm against lettuce seedlings.^104^ However, a recent study showed that (+)-streptol (**29**) is not toxic to plant aerial organs and does not primarily impact cell proliferation or root patterning.^49^ The compound appears to disturb root growth anisotropy at the onset of cellular elongation by interfering with primary cell wall biogenesis or remodeling processes in a cell type- or tissue-specific manner.^49^ It directly or indirectly disturbs root cell wall organization without affecting cellulose biosynthesis. While the exact mechanism of this interesting biological activity is currently unknown, several hypotheses exist. Given that (+)-streptol (**29**) is structurally similar to glucose, it may interfere with glucose-processing enzymes.^49^ Some cyclitol kinases and nucleotidyltransferases may be able to convert (+)-streptol (**29**) to its nucleotidyl derivative,^105–107^ which is then incorporated into one of the major carbohydrate polymers of plant cell walls. Finally, the possibility that (+)-streptol (**29**) and/or its derivatives function as signaling molecules (similar to *myo*-inositols) cannot be ruled out.

Comparisons of the root growth inhibitory activity between (+)-streptol, (+)-streptol β-glucoside, and A-79197-2 [(+)-streptol α-glucoside] revealed that (+)-streptol (IC_50_ 17 μM) was slightly more active than (+)-streptol β-glucoside (IC_50_ 28 μM), whereas A-79197-2 (IC_50_ > 100 μM) was significantly less active than the other two compounds.^108^ A similar study using the same set of compounds against *Arabidopsis* gave IC_50_ values of 4.8 μM, 9.4 μM, and 17 μM, respectively. While the IC_50_ values of (+)-streptol β-glucoside (**30**) were somewhat higher than those of (+)-streptol (**29**), it remains to be determined if glycosylation of (+)-streptol is intended to protect the host plant *P. kirkii* from its growth inhibitory effects. In fact, (+)-streptol (**29**) or *P. kirkii* extracts can inhibit root growth anisotropy (at the onset of cellular elongation) of many other plants including *Arabidopsis*, but not that of the host plant *P. kirkii*.^49^ *P. kirkii* was the only seed plant that was found to be insensitive to (+)-streptol, leading to the hypothesis that (+)-streptol is used during competition for space and nutrients.^104^ Soaking *Arabidopsis* roots in a solution of 250 μM pure (+)-streptol glucoside (**30**) for 48 h followed by careful washing and analysis only gave (+)-streptol (**29**) (4.8 ± 0.3 μM), but not (+)-streptol glucoside (**30**), in the plant. Therefore, it was suggested that (+)-streptol glucoside (**30**) acts as a prodrug, in which the glucose unit is necessary for uptake but then hydrolyzed in the plant to a more active compound, (+)-streptol (**29**).

Interestingly, the C_7_N-aminocyclitol kirkamide (**6**) and the C_7_-cyclitols (+)-streptol (**29**) and (+)-streptol β-glucoside (**30**) are all produced by “*Ca*. B. kirkii”, but they appear to have two distinct biological activities (Figure 10).^108^ While kirkamide (**6**) protects the host plant from insects, (+)-streptol (**29**) and (+)-streptol β-glucoside (**30**) assist the plant to compete with other plants for space and nutrients.^48,108^ On the other hand, genomic evidence points to a single cyclitol forming BGC, indicating that the biosynthetic pathway is adaptable, producing different compounds depending on the signals and/or the needs of the host organism.^100^

### Mycosporine-like Amino Acids and Their Signaling Activity

Another group of C_7_N-aminocyclitols, known as mycosporine-like amino acids (MAAs), has been identified in bacteria, cyanobacteria, fungi, phytoplankton, macroalgae, and marine animals.^109–115^ These water-soluble, colorless small molecules are characterized by their ability to absorb ultraviolet (UV) light typically between 310 and 340 nm, suggesting their photoprotective benefit to the producing organisms. Although earlier studies refer mostly to this UV-protective ability, scientific evidence suggests that MAAs have other functional roles, including antioxidant activity and osmotic regulation.^116,117^ Their antioxidant and free radical scavenging activity can prevent cellular damage caused by UV-induced reactive oxygen species (ROS).^116^ Consequently, MAAs have attracted significant research attention from academia and industry alike. More recently, reports on MAAs’ effects on gene expression and signaling pathways in human cells have begun to emerge. Some major MAAs, *i.e*., porphyra-334 (**8**), shinorine (**31**), and mycosporine-glycine (**32**) (Figures 1 and 11), have been shown to promote wound healing process in human keratinocytes through activation of focal adhesion kinases (FAK), extracellular signal-regulated kinases (ERK), and c-jun N-terminal kinases (JNK), suggesting that they accelerate wound healing by activating the FAK-MAPK signaling pathways (Table 1).^118^ Porphyra-334 (**8**) was also found to accelerate somatic cell reprogramming of mouse tail-tip fibroblasts and human dermal papilla cells into induced pluripotent stem cells.^119^ Specifically, it accelerates the mesenchymal to epithelial transition (MET) process in cell programming by activating the Histone H3 Lysine 4 trimethylation and controlling histone modification.

**Table 1:** Reported Signaling Activities of the Mycosporine-like Amino Acid Porphyra-334 (**8**)

Signaling activity	Potential outcome	References
Activate FAK-MAPK signaling pathways	Increase wound healing process	^118^
Activate the H3K4me3 and histone modification	Accelerate somatic cell reprogramming	^119^
Attenuate the caspase pathway	Inhibit UV-induced apoptosis	^51^
Increase the Wnt and the Notch signaling pathways	Increase responses to UV irradiation	^52^
Regulate the expression of certain miRNAs	Regulate UV-mediated cellular processes	^52^
Promote collagen synthesis in fibroblast cells	Delay aging	^50^
Upregulate the expression of metallothionein-associated genes	Improve metal homeostasis and reduce oxidative stress	^50^
Upregulate genes associated with hair follicle cycle and epidermal structure	Improve hair follicle growth and maintenance	^50^

**Figure 11:**
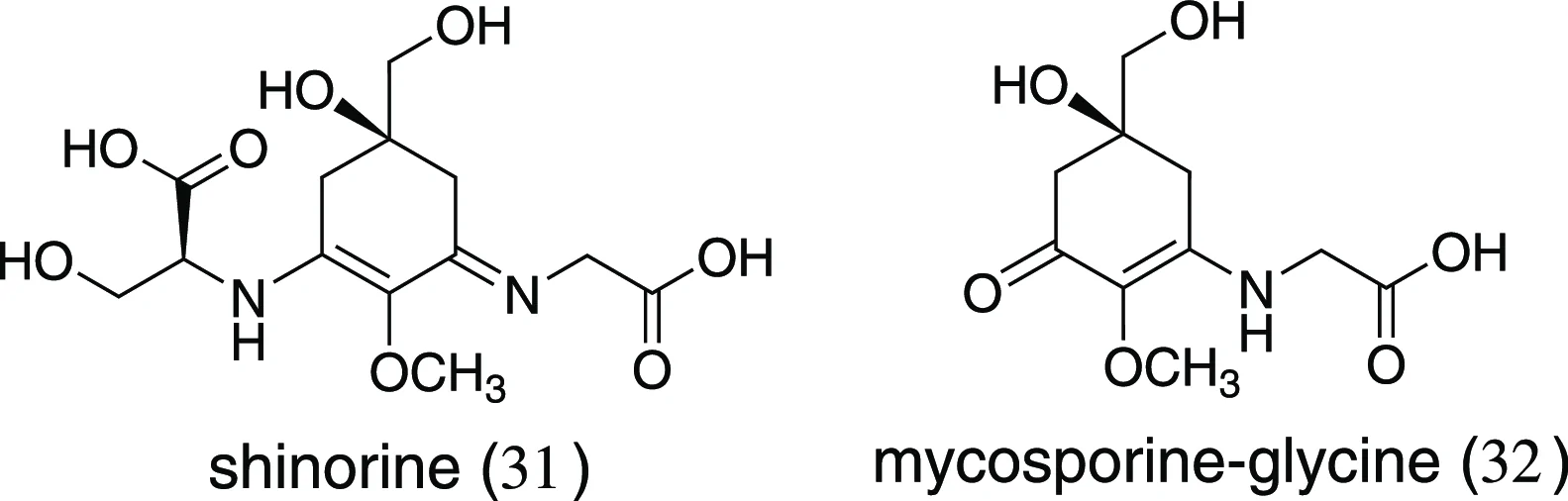
Chemical structures of major mycosporine-like amino acids (MAAs).

Furthermore, porphyra-334 (**8**) has been shown to inhibit UV-induced apoptosis in human immortalized keratinocyte (HaCaT) cells through attenuation of the caspase pathway.^51^ Expression of active caspase-3 protein in HaCaT cells was increased in response to UV irradiation, whereas cells treated with porphyra-334 and irradiated with UV light showed similar expression levels with the nonirradiated control group. Subsequently, gene expression profiling using cDNA and miRNAs microarray in HaCaT cells exposed to UV irradiation revealed the beneficial effects of porphyra-334 on the Wnt (Wingless/integrase-1) and the Notch signaling pathways in respond to the UV radiation.^52^ Porphyra-334 also regulates the expression of certain miRNAs that target genes related to UV-mediated cellular processes, including apoptosis, cell proliferation, and translational elongation (Table 1).^52^ The data suggest that the UV protective effects of MAAs are not solely due to their UV absorption and antioxidant activities, but are orchestrated through a comprehensive modulation of gene expression and regulation in the cells in response to UV irradiation.

A more recent study also showed some evidence of porphyra-334 (**8**) as a potential antiaging agent.^120^ Porphyra-334 (**8**) was found to promote collagen synthesis in fibroblast cells in a dose dependent manner.^50^ It was also linked to the improvement of periorbital wrinkles in a clinical test with human participants.^50^ It upregulated the expression of metallothionein-associated genes, which play crucial roles in metal homeostasis and oxidative stress protection. In addition, porphyra-334 upregulated genes involved in nuclear chromosome segregation and those that encode components of kinetochores in human follicle dermal papilla (HFDP) cells.^50^ It also upregulated several genes associated with the hair follicle cycle, the hair follicle structure, and the epidermal structure, suggesting the potential role of porphyra-334 in hair follicle growth and maintenance.^50^ While MAAs are produced mostly by bacteria, cyanobacteria, fungi, phytoplankton, and macroalgae, there is plenty of evidence that they are obtained and accumulated by higher order animals such as fish and marine invertebrates.^109,121,122^ However, it is unclear whether MAAs act as signaling molecules or provide other benefits for the organisms beyond the conventional belief of their UV-protection.

Extracts of *Porphyra tenera* that contain porphyra-334 (**8**) have also been reported to modulate cognitive and intestinal functions in particulate matter (PM_2.5_)-induced cognitive decline in mice (Figure 12).^123^ They attenuated learning and memory impairment through antioxidant and anti-inflammation effects by regulating the mitochondrial function and TLR-initiated NF-κB signaling. The extracts also reduced the expression of the cognitive-related molecule amyloid beta (Aβ) and tau phosphorylation. While the extracts showed beneficial effects on cognitive and intestinal functions in mice, further studies are needed to determine whether MAAs play a significant role in the observed activities. Also, it is important to note that the various effects of MAAs summarized above may require additional or more in-depth studies to interpret and possibly link some of the observed signaling activities and cellular responses.

**Figure 12:**
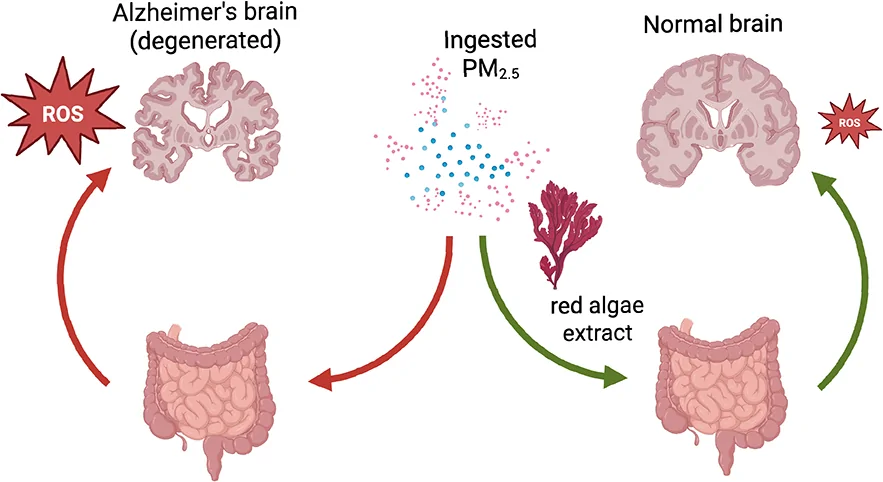
Extracts of *Porphyra tenera* can modulate cognitive and intestinal functions in particulate matter (PM_2.5_)-induced cognitive decline mice (Created in BioRender. Hennelly, T. (2026) https://BioRender.com/xm0yo6s).

## The Potential Regulatory Roles of the Cryptic Ebo Clusters

The ebo (for “eustigmatophyte-bacterial operon”) cluster of genes (*eboA*–*eboF*) is found in a variety of genomic contexts among diverse organisms, including Gram-(−) bacteria (*e.g*., acidobacteria and cyanobacteria) and eustigmatophyte algae (*e.g*., *Monodopsis* sp., *Vischeria* sp.) (Figure 13A).^22–25^ The cluster contains genes predicted to encode proteins similar to 3-dehydroquinate synthase (DHQS-like, EboD) and a UbiA-like prenyltransferase (EboC), as well as four proteins of unknown functions, but are distantly similar to sugar phosphate isomerases/epimerases, metal-dependent hydrolases, and phosphodiesterases. EboD was predicted to be a novel variant of DHQS, which catalyzes the cyclization of DAHP to DHQ in the shikimate pathway. Later, this protein was found to be a new member of the sugar phosphate cyclase family, catalyzing the cyclization of mannose 6-phosphate to a novel C_6_-cyclitol, 2-deoxy-4-*epi*-*scyllo*-inosose (DEI) (Figure 2). While the product of the ebo cluster is still unknown, in *Nostoc punctiforme*, this cluster has been proposed to be involved in the transport of the precursor of the sunscreen compound scytonemin from the cytosol to the periplasm (Figure 13B).^22^ However, how exactly this cluster is involved in the transport of scytonemin monomers from the cytosol to the periplasm is unknown.

**Figure 13:**
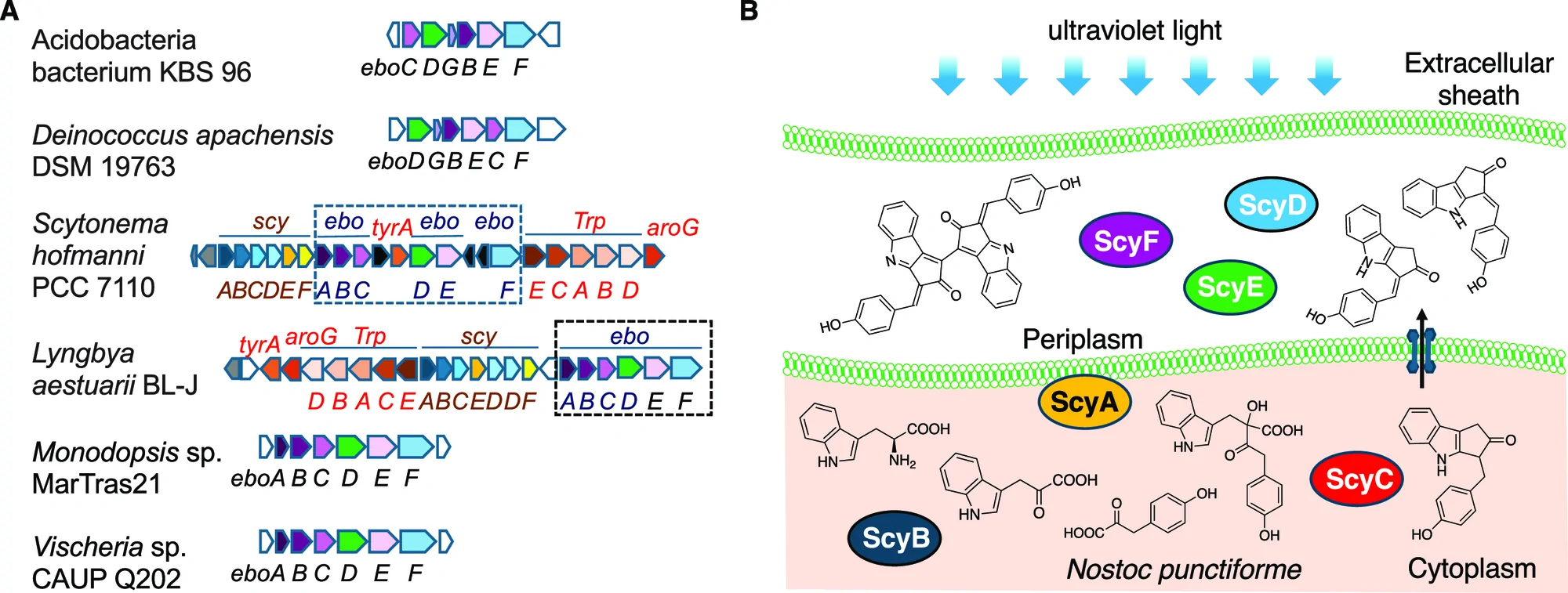
Ebo cluster in Gram-(−) bacteria and eustigmatophyte algae. (A) The ebo cluster in various Gram-(−) bacteria (*e.g*., acidobacteria and cyanobacteria) and eustigmatophyte algae. In cyanobacteria, the ebo cluster (in dashed boxes) is typically located next to the scytonemin gene cluster; (B) the ebo cluster has been reported to play a role in the translocation of scytonemin monomers from the cytoplasm to the periplasm of cyanobacteria. The final oxidative dimerization step takes place in the periplasm.

Interestingly, a variant of the ebo cluster, known as the EDB (for “edible”) cluster, is necessary for *Pseudomonas fluorescens* NZI7 to repel grazing by the nematode *C. elegans*.^25^ The EDB cluster contains six genes homologous to those of the ebo cluster plus three additional genes with unknown function. Inactivation of genes homologous to *eboD* and *eboC* independently in *P. fluorescens* NZI7 resulted in mutants that are no longer able to repel *C. elegans*.^26^ The study also showed that the EDB cluster is involved in regulating tryptophan catabolism to produce (*1H*-indol-3-yl)-oxoacetamide and indole-3-aldehyde, and that these products were found to be sufficient to repel *C. elegans* (Figure 14).^26^ Heterologous expression of the NZI7 EDB cluster in *E. coli* resulted in the production of a large amount of tryptophol and several other indole derivatives, including novel indole dimeric compounds.^26^ Some human gut microbiota (*e.g*., acidobacteria) harbor EDB, and tryptophan catabolites from gut microbes influence human health.^124^ Tryptophol is also a fungal quorum sensing molecule that can induce programmed cell death and prevent biofilm formation.^125,126^ Despite its potential benefits for human health, the product of the EDB cluster is currently unknown. Nevertheless, as the indole derivatives are less likely to be the products of the EDB cluster, it may be assumed that the EDB cluster plays a role in regulating their formation, either directly (*e.g*., regulating the catabolic enzyme expression) or indirectly (*e.g*., functions as a cofactor or a transporter for the indoles or the processing enzymes). Hence, characterization of the EDB product is important, as it may accelerate progress toward understanding its mechanism in inducing tryptophan catabolism and its potential benefits for human health.

**Figure 14:**
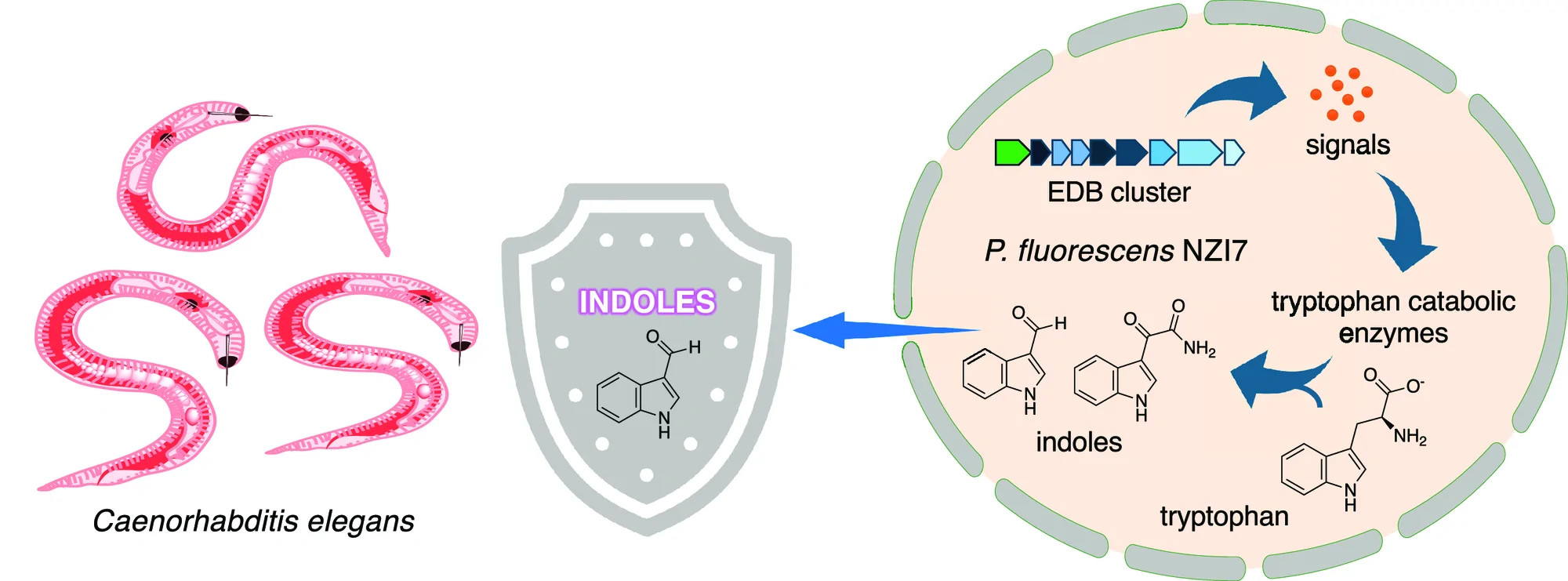
EDB cluster is involved in the ability of *Pseudomonas fluorescens* NZI7 to repel the nematode *C. elegans*. It is proposed that the cluster produces a molecule that is directly or indirectly involved in tryptophan catabolism, converting tryptophan to (*1H*-indol-3-yl)-oxoacetamide and indole-3-aldehyde, which are sufficient to repel *C. elegans*.^25,26^

## Summary and Perspectives

Naturally occurring cyclitols and aminocyclitols are known to have a variety of biological activities, and some of them may function as signals. Inositol and its derivatives are the prime examples of this, as they orchestrate the spatial and temporal regulation of many signaling cascades contributing to early life development as well as playing a role in metabolic homeostasis and pathogenesis in humans, animals, and plants.^27–30^ In nature, inositols also play important ecological roles, such as in nutrient cycles and in plant’s host–endophyte interactions.^31,32^ Other cyclitols and aminocyclitols have also been reported to function as signals that contribute to various biological processes and homeostasis of ecosystems. This includes the role of aminoglycoside antibiotics in regulating biofilm formation in bacteria as well as inducing secondary metabolite production in fungi.^33–36^ However, studies also showed that sub-MIC doses of the aminoglycoside streptomycin are sufficient to inhibit competing susceptible bacterial cells and to provide natural selection to enrich for resistance.^78^ Therefore, whether antibiotics in the environment are really used as signals or as weapons or both remains a matter of discussion.

Members of the C_7_-cyclitol/C_7_N-aminocyclitol family of natural products, *e.g*., acarbose, validamycin A, and streptol, have been associated with human gut microbiota modulation, gene regulation, and/or symbiosis, suggesting involvement of signaling. However, some of their exact mechanisms are not completely understood. Therefore, more detailed studies are needed to understand their actual roles in each of those processes. While mycosporine-like amino acids (MAAs) are well-known for their UV-protective activities, studies with human cells and animals have revealed intriguing biological activities. Many of them involve gene regulation and activation of signaling pathways that result in various benefits, ranging from wound healing to delaying aging, to improving hair follicle growth. Since MAAs are also accumulated in fish and marine invertebrates, most likely from their diet, it may be speculated that MAAs provide other benefits to those animals beyond their potent UV-protective activity.

In conclusion, there is a growing body of evidence to suggest that some naturally occurring cyclitols and aminocyclitols are being used by the producing organisms as signals, which in the environment play a significant role in the well-being of the producers and maintaining the homeostasis of ecosystems. Some of them may have additional biological activities by affecting cellular signaling pathways, gene expression, and microbiota, which may provide significant benefits to humans, animals, and plants. However, further studies are needed to understand their roles in nature and their various benefits to humans. Also, beyond the currently known cyclitol/aminocyclitol natural products are the presence of numerous cryptic biosynthetic gene clusters (BGCs) in the genome sequence databases with putative novel cyclitol/aminocyclitol products. Investigating those cryptic BGCs will provide new opportunities for discovering new natural products and understanding their biological roles in the environment and in improving human health.
